# Treprostinil stimulates the engraftment of haematopoetic stem cells

**DOI:** 10.1186/1471-2210-11-S2-A6

**Published:** 2011-09-05

**Authors:** Filza Hussain, Christian Bergmayr, Armam Alimohammadi, Michael Freissmuth, Eva-Maria Zebedin

**Affiliations:** 1 Institute of Pharmacology, Center of Physiology and Pharmacology, Medical University of Vienna, 1090 Vienna, Austria

## Background

Successful transplantation of haematopoetic stem cells (HSC) is often limited by low transplantation efficiency. This may be enhanced by pharmacological means. In fact, HSCs require a Gα_s_-transduced signal to re-populate the bone marrow [1]. Pretreatment with prostaglandin E_2_ (PGE_2_) enhances engraftment via activation of Gα_s_-coupled EP_2_ and EP_4_ receptors [2]. Treprostinil is a stable analogue of prostacyclin/PGI_2_. It predominantly acts via EP_2_ and EP_4_ receptors. Treprostinil is approved for treatment of pulmonary hypertension. Here we test the hypothesis that treprostinil may also be useful to promote engraftment of HSC.

## Methods

Generation of murine bone marrow-derived HSCs: Undifferentiated HCS (lineage-negative, Lin^−^ cells) were separated from bone marrow cells by MACS (magnetic-assisted cell sorting) and characterized by fluorescence-activated cell sorting (FACS) using cell surface markers. [^3^H]cAMP-accumulation assays: Lin^−^ cells were incubated in supplemented stem cell medium (StemSpanSFEM#09650). After 4 h at 37°C cells were stimulated with forskolin, treprostinil, other prostanoids and cholera toxin for 1 h. Bone marrow transplantation: Recipient mice were lethally irradiated. Lin^−^ cells were pretreated in absence/presence of 10 µM treprostinil, treprostinil plus 30 µM forskolin or 10 µg/mL cholera toxin for 1 h at 37°C. 3x105 cells/mouse were injected via the tail vein. Transplantation efficiency was determined by the analysis of white blood cell counts. For competition assay, equal numbers of treated/untreated Lin^−^ cells, which can be distinguished according to surface expression of Ly5.1 and Ly5.2, were injected in one and the same recipient mouse.

## Results

Successful MACS-purification of Lin^−^ cells was documented by FACS. Next, the cAMP-response of Lin^−^ cells to treprostinil was tested: Treprostinil elicited a concentration-dependent accumulation of cAMP in the range of 0.1–10 µM with an estimated EC_50_ in the range of 0.3 µM. A beneficial effect was also observed *in vivo:* mice injected with treprostinil-pretreated Lin^−^ cells had significantly higher levels of circulating white blood cells when compared to those receiving vehicle-treated Lin^−^ cells (p < 0.05, unpaired *t* test). In addition, when pretreated and untreated Lin^−^ cells were mixed to compete for bone marrow reconstitution, the white blood cells derived from the pretreated Lin^−^ cell population were 1.5–3-fold more abundant than those originating from the non-treated HSC.

## Conclusions

The treprostinil-induced cAMP elevation translates into enhanced engraftment of haematopoetic stem cells. Because treprostinil is reasonably well tolerated, it may be of interest to explore its action in bone marrow transplantation in people.
